# Congenital and Neonatal Malaria in Ethiopia: A Narrative Review

**DOI:** 10.1155/japr/6152607

**Published:** 2026-04-15

**Authors:** Yemane Leake, Hindeya Hailu Hagos, Birhanu Kassie Reta

**Affiliations:** ^1^ Department of Pediatrics and Child Health, College of Health Sciences, Aksum University, Aksum, Ethiopia, aku.edu.et; ^2^ Department of Pathology, College of Health Sciences, Aksum University, Aksum, Ethiopia, aku.edu.et

**Keywords:** congenital malaria, Ethiopia, malaria, narrative review, neonatal malaria, neonate

## Abstract

**Background:**

Malaria remains a major public health problem in Ethiopia. Congenital malaria results from transplacental transmission of malaria parasites from mother to fetus before or during delivery, whereas neonatal malaria is acquired after birth, typically through mosquito bites within the first 28 days of life. Both conditions are associated with significant neonatal morbidity and mortality in endemic settings. Despite Ethiopia′s high malaria burden, evidence on congenital and neonatal malaria remains fragmented and limited.

**Objective:**

This narrative review synthesizes the existing literature on congenital and neonatal malaria in Ethiopia, highlighting epidemiology, clinical presentation, diagnosis, management, and prevention, with the aim of informing clinical practice and guiding future research.

**Methods:**

Published studies reporting congenital and neonatal malaria in Ethiopia were identified through searches of PubMed, Google Scholar, Medline, and grey literature sources. Evidence was synthesized narratively, with emphasis on patterns, themes, and clinical implications. All eligible studies identified through PRISMA‐guided screening were incorporated into the synthesis.

**Results:**

Ten studies conducted in Ethiopia reported a total of 46 cases of congenital and neonatal malaria. *Plasmodium falciparum* was the predominant species (35 cases), followed by *Plasmodium vivax* (eight cases) and mixed infections (three cases). Forty cases were classified as congenital malaria, whereas six were neonatal malaria. Most reports were case reports or case series, reflecting the limited scope of available data.

**Conclusion:**

Congenital and neonatal malaria in Ethiopia are under recognized despite the country′s substantial malaria burden. *P. falciparum* is the dominant causative species. Greater clinical awareness, improved diagnostic vigilance, and larger epidemiological studies are urgently needed to better define disease burden and optimize prevention and management strategies for this vulnerable population.

## 1. Background

Malaria remains a major public health challenge globally and continues to cause substantial morbidity and mortality in sub‐Saharan Africa. According to the World Health Organization (WHO) World Malaria Report 2024, Ethiopia contributed significantly to the recent global increase in malaria cases, with an estimated 59% rise in national malaria incidence between 2022 and 2023 [1]. Achieving global malaria reduction targets requires effective prevention, early diagnosis, and timely treatment across all age groups, including neonates [2].

Studies on congenital and neonatal malaria have reported widely variable findings both globally and across Africa. For example, a systematic review from Nigeria showed that the prevalence of congenital malaria differed markedly between studies, ranging from 5.1% to 96.3% [3]. Similarly, a meta‐analysis reported an overall prevalence of 40.4%, with substantial heterogeneity across studies, and found no significant difference between prevalence estimates from Africa (39.5% and those from regions outside Africa 56.3% [4].

Ethiopia is classified as a high‐burden malaria country, with approximately 52% of its population living in malaria‐endemic areas and nearly three‐quarters of the country considered malarious [5]. *Plasmodium falciparum* and *Plasmodium vivax* are the predominant species, accounting for about 60% and 40% of infections, respectively.

While malaria in older children and adults has been extensively studied, malaria occurring during the neonatal period has received far less attention. Congenital malaria results from vertical transmission of parasites from mother to fetus during pregnancy or delivery, whereas neonatal malaria is acquired after birth, typically through mosquito bites within the first 28 days of life [6]. Both conditions may lead to severe illness and death if unrecognized. This narrative review synthesizes available evidence on congenital and neonatal malaria in Ethiopia, focusing on epidemiology, clinical presentation, diagnosis, management, and prevention.

## 2. Methods and Materials

A narrative review approach was used to summarize existing literature on congenital and neonatal malaria in Ethiopia. Relevant publications were identified through searches of PubMed, MEDLINE, Google Scholar, and grey literature sources using combinations of terms related to malaria, neonates, congenital infection, and Ethiopia. Case reports, case series, and observational studies were considered.

Rather than applying a rigid systematic‐review methodology, the evidence was synthesized descriptively, with emphasis on recurring themes, clinical patterns, diagnostic challenges, and implications for practice.

## 3. Study Selection and Data Extraction

Articles were initially screened based on titles and abstracts. Full‐text articles were retrieved when the abstract did not provide sufficient information. Screening criteria included relevance to congenital or neonatal malaria, English language, participant age (neonates), and study location (Ethiopia).

**FIGURE 1:**
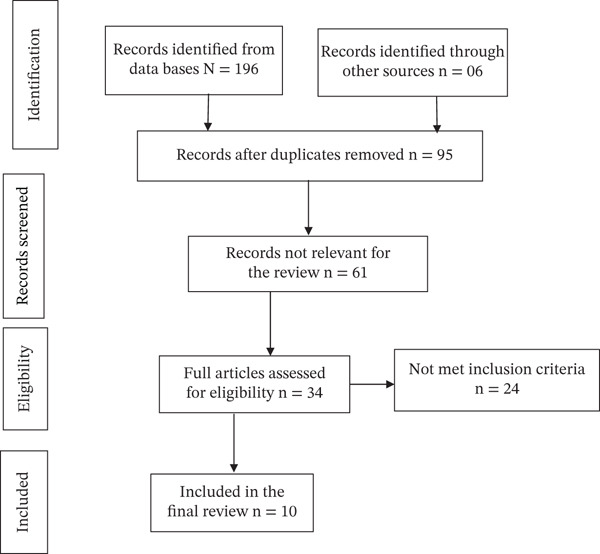
PRISMA 2020 flow diagram illustrating the study selection process for the narrative review.

The search identified 202 studies from electronic databases. After removing duplicates and excluding irrelevant articles based on title and abstract, 34 full‐text articles were assessed. Of these, 10 studies were included in the final review, whereas the remaining 24 articles were excluded due to inadequate information (Figure 1). Discrepancies were resolved by discussion among the reviewers.

## 4. Results

### 4.1. Epidemiology and Species Distribution

Ten studies [7–16] conducted in Ethiopia reported a total of 46 cases of congenital and neonatal malaria (Table 1).

**TABLE 1:** Characteristics of studies reporting congenital and neonatal malaria in Ethiopia.

Author, year	Study population	CM vs. NM	Sample size	Study area	Study design	Malaria species (P.f/P.v/mixed)
Kebedom et al., 2020 [7]	Neonate	CM	1	Afar Region	Case report	1/0/0
Solomon et al., 2020 [8]	Neonate	CM	4	Central, Wolkite	Article	4/2/0
Tamir et al., 2023 [9]	Neonate	CM	15	North West, Jawi District	Article	15/2/0
Tesso et al., 2024 [10]	Neonate	NM	3	Southern Ethiopia	Case series	0/0/0
Tessema et al., 2024 [16]	Neonate	NM	1	Southern Ethiopia	Case report	0/1/0
Regasa et al., 2024 [11]	Neonate	NM	1	Western Ethiopia	Case report	0/1/0
Gedefaw et al., 2024 [12]	Neonate	CM	1	Northwest Ethiopia	Case report	0/1/0
Alemayehu et al., 2024 [13]	Neonates	CM	9	Gambella Region	Article	8/1/0
Gebremichael et al., 2025 [14]	Neonate	CM/NM	6	Northern Ethiopia	Case series	4/1/1
Andarge and Almaw, 2025 [15]	Neonate	CM	1	Southern Ethiopia	Case report	0/1/0

Abbreviations: CM = congenital malaria, NM = neonatal malaria; P.f = *Plasmodium falciparum*; P.v = *Plasmodium vivax.*

**FIGURE 2:**
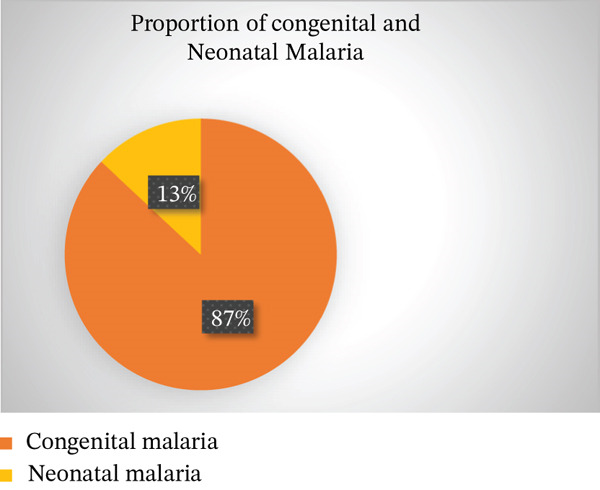
Proportion of congenital versus neonatal malaria cases in Ethiopia, a pie chart showing the proportion of congenital malaria (CM) and neonatal malaria (NM) among the 46 cases reported in the included studies. Congenital malaria accounted for 87% of cases, whereas neonatal malaria represented 13%.

**FIGURE 3:**
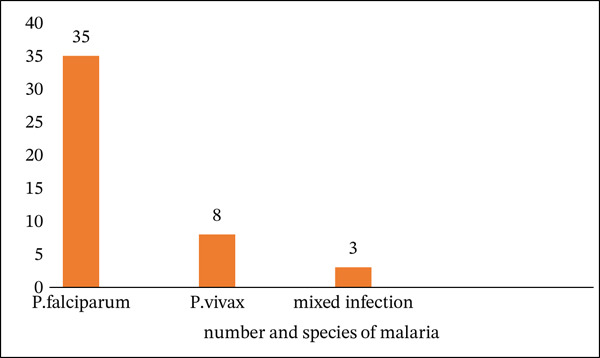
Distribution of Plasmodium species causing congenital and neonatal malaria in Ethiopia, a bar chart showing the number of cases caused by *Plasmodium falciparum*, *Plasmodium vivax*, and mixed infections across the 10 included studies. *P. falciparum* was the predominant species, followed by *P. vivax* and mixed infections.

Forty cases were classified as congenital malaria, whereas six were neonatal malaria (Figure 2). *P. falciparum* was the predominant species (35 cases), followed by *P. vivax* (eight cases) and mixed infections (three cases) (Figure 3). Most studies were case reports or case series, reflecting the limited scope of available data.

Placental malaria prevalence is high in endemic areas, with studies reporting up to 34.4% placental infection among pregnant women in regions such as Gambella [14], suggesting widespread fetal exposure.

### 4.2. Clinical Presentation

Fever was the most frequently reported symptom among neonates with congenital or neonatal malaria [3, 17]. In several cases, fever appeared within the first days of life, prompting malaria testing. Nevertheless, clinical presentation was often nonspecific. Neonates also presented with poor feeding, irritability, pallor, jaundice, abdominal distension, anemia, thrombocytopenia, hypoglycemia, respiratory distress, hepatosplenomegaly, or convulsions [18–20]. Afebrile presentations were reported in southern Ethiopia, with pallor, anemia, and splenomegaly as predominant features [10]. Table 2 summarizes the main clinical presentations and physical findings.

**TABLE 2:** Summary of reported clinical presentations of neonatal and congenital malaria in Ethiopia.

Author, year	Main clinical presentations and physical findings
Kebedom et al., 2020 [7]	High grade fever, decreased suckling and weight loss
Solomon et al., 2020 [8]	No records
Tamir et al., 2023 [9]	No records
Tesso et al., 2024 [10]	Vomiting, refusal to feed, pallor, severe anemia, splenomegaly, inconsolable cry, failure to pass feces and abdominal distension
Tessema et al., 2024 [16]	High grade fever, abnormal body movement ,tachypnea and tachycardia
Regasa et al., 2024 [11]	Vomiting, high grade fever and splenomegaly
Gedefaw et al., 2024 [12]	Fever, decreased mentation, decreased sucking , abnormal body movements, palpable spleen and liver, not sustained sucking and Moro reflexes
Alemayehu et al., 2024 [13]	No records
Gebremichael et al., 2025 [14]	High grade fever, vomiting ,altered mental status, abnormal body movement , irritability and excessive/intense cry, breastfeeding refusal , decreased urine amount, weight loss, jaundice, depressed primitive reflexes, hypoglycemia, anemia and thrombocytopenia
Andarge and Almaw, 2025 [15]	Respiratory distress, persistent fever

### 4.3. Diagnostic Challenges

Neonatal malaria often mimics neonatal sepsis, making early recognition particularly challenging in clinical practice. Both conditions share overlapping and nonspecific symptoms, including fever, vomiting, abdominal distension, poor or reduced feeding, irritability, hypoglycemia, and anemia [19]. These similarities frequently lead clinicians to initially suspect neonatal sepsis, which is more commonly encountered and widely recognized. As a result, most neonates in the reviewed studies were empirically treated with broad‐spectrum antibiotics at presentation. In many cases, malaria was considered only after the persistence of symptoms despite appropriate antibiotic therapy, lack of clinical improvement, or identification of risk factors such as maternal malaria, residence in endemic areas, or unexplained anemia and thrombocytopenia. This delay in considering malaria as a differential diagnosis can contribute to prolonged illness, delayed initiation of appropriate antimalarial therapy, and increased risk of complications.

The diagnosis of neonatal malaria primarily relies on microscopic examination of thick and thin peripheral blood smears, which remains the gold standard due to its ability to detect and quantify parasitemia and identify the Plasmodium species [21]. However, the sensitivity of microscopy may be reduced in neonates because parasite densities are often low, particularly in congenital malaria, and detection depends heavily on the expertise of laboratory personnel. Rapid diagnostic tests (RDTs), which detect specific malaria antigens, offer a useful alternative, especially in settings where microscopy expertise is limited, and they provide quicker results. Additionally, molecular methods such as polymerase chain reaction (PCR) offer superior sensitivity and specificity, enabling detection of low‐level parasitemia and confirmation of species. Despite these advantages, the use PCR in Ethiopia remains limited due to infrastructure constraints, high costs, limited availability of specialized equipment, and lack of trained personnel. Strengthening laboratory capacity and increasing awareness among healthcare providers are essential to improve early diagnosis and appropriate management of neonatal malaria in endemic settings.

### 4.4. Treatment and Management

Neonates have generally been excluded from clinical trials, and the WHO has not formulated specific treatment guidelines for malaria in this age group. Consequently, antimalarial medications are often used off‐label in neonates, with dosing typically extrapolated from the recommended milligram/kilogram schedules for older children. Intravenous artesunate (3 mg/kg per dose) was the most commonly used therapy [21]. Treatment duration ranged from three doses(at 0, 12, and 24 h) of artesunate to 7 days (Table 3). One of the studies reported that they transitioned to oral artemether–lumefantrine after initial stabilization with artesunate [15]. Challenges included lack of neonatal‐specific formulations, resulting in potential under‐ or overdosing. Primaquine should be avoided in neonates due to safety concerns [21, 22]. Neonatal pharmacokinetics and pharmacodynamics differ from older children, complicating therapy.

**TABLE 3:** Summary of reported treatments for neonatal and congenital malaria in Ethiopia.

Author, year	Study population	Treatment	Duration of treatment
Kebedom et al., 2020 [7]	Neonate	Artesunate with unspecified dose	For 3 days
Solomon et al., 2020 [8]	Neonate	No records	
Tamir et al., 2023 [9]	Neonate	No records	
Tesso et al., 2024 [10]	Neonate	Artesunate 3 mg/kg	For 7 days
Tessema et al., 2024 [16]	Neonate	Artesunate 3 mg/kg	For 7 days
Regasa et al., 2024 [11]	Neonate	Artesunate 3 mg/kg	For 5 days
Gedefaw et al., 2024 [12]	Neonate	Artesunate 3 mg/kg	For 4 days
Alemayehu et al., 2024 [13]	Neonates	No records	
Gebremichael et al., 2025 [14]	Neonate	Artesunate 3 mg/kg	For 5–7 days
Andarge and Almaw, 2025 [15]	Neonate	Artesunate 3 mg/kg for three doses	Transitioned to oral artemisinin–lumefantrine for 3 days

*Note:* The first three doses of artesunate are given at 0, 12, and 24 h for all cases.

## 5. Discussion

This literature review synthesizes the available evidence on congenital and neonatal malaria in Ethiopia, providing a comprehensive overview of epidemiology, clinical presentation, diagnosis, management, and prevention. The review was conducted following PRISMA (Preferred Reporting Items for Systematic Reviews and Meta‐Analyses) guidelines to ensure a systematic, transparent, and reproducible approach. A total of 10 studies conducted in Ethiopia, including case reports and case series, were identified and analyzed as the primary database. By consolidating these limited and fragmented reports, this review addresses a critical knowledge gap in neonatal malaria, a condition that is often underrecognized despite its potentially severe outcomes. The findings aim to support clinicians in the early recognition and timely management of affected neonates, guide policymakers and public health programs in designing targeted interventions, and stimulate further research to improve understanding and outcomes for this neglected yet life‐threatening condition in Ethiopia.

The epidemiology of congenital and neonatal malaria appears to be influenced by the level of malaria endemicity. Vertical transmission is thought to occur less frequently in highly endemic areas, where maternal immunity is more robust, and more commonly in areas of lower or unstable transmission [6]. Placental malaria, however, remains common even among asymptomatic mothers in endemic regions. Studies have reported placental infection in up to one‐third of pregnant women, including a report from the Gambella region of Ethiopia that documented a prevalence of 34.4% [13] . These findings suggest that fetal exposure to malaria parasites may be more common than clinically apparent neonatal disease.

Regarding clinical presentation, the reviewed literature indicates that fever emerges as the most frequently reported symptom among neonates with congenital and neonatal malaria. Several reports describe fever appearing as early as the first day of life, often prompting clinicians to initiate further investigations. Similar observations have been reported from other malaria‐endemic countries. For example, a review from Nigeria identified fever as a nearly universal feature of congenital malaria, and case series from Uganda also documented fever in most affected neonates [3, 17]. These findings underscore the importance of maintaining a high index of suspicion for malaria when evaluating febrile neonates in endemic areas.

Nevertheless, congenital and neonatal malaria often present with nonspecific clinical features that overlap with other neonatal conditions. Reported manifestations include poor feeding, jaundice, excessive crying, vomiting, diarrhea, convulsions, anemia, thrombocytopenia, hypoglycemia, respiratory distress, hepatosplenomegaly, hypothermia, apnea, and, in some cases, an absence of symptoms altogether [18–20]. Notably, a case series from southern Ethiopia described neonates who presented without fever, with pallor, abdominal distension, anemia, and splenomegaly as the predominant features [10]. Such atypical or afebrile presentations further complicate early recognition.

A recurring challenge highlighted in the literature is the clinical resemblance between neonatal malaria and neonatal sepsis. Both conditions share overlapping symptoms and laboratory abnormalities, including fever, vomiting, abdominal distension, reduced feeding, irritability, hypoglycemia, and anemia [19]. As a result, neonates with malaria are frequently treated empirically for sepsis at presentation. In many reports, persistence of fever or lack of clinical improvement despite antibiotic therapy ultimately prompted malaria testing, leading to the diagnosis. This diagnostic overlap emphasizes the need to consider malaria in the differential diagnosis of neonatal sepsis, particularly in endemic settings.

Several protective mechanisms may limit the clinical expression of malaria in neonates despite parasitemia. These include passive transfer of maternal antibodies, the predominance of fetal hemoglobin, which is less favorable for parasite growth, and the composition of breast milk, which is relatively low in iron and p‐aminobenzoic acid—both essential for parasite replication [23, 24]. These factors may explain why some neonates remain asymptomatic or present with delayed or mild disease.

Diagnosis of congenital and neonatal malaria relies primarily on parasitological confirmation. Most reports describe the use of thick and thin blood smears, which remain the diagnostic standard, although low parasite densities in neonates may reduce sensitivity [23]. RDTs and molecular methods such as PCR can improve detection, particularly in low‐parasitemia cases. However, access to PCR remains limited in low‐resource settings, including Ethiopia, due to cost and infrastructure constraints.

Treatment approaches for congenital and neonatal malaria generally mirror those used in older children, with adjustments for age and clinical severity. Parenteral therapy is often preferred in neonates because of the risk of rapid deterioration and challenges with oral drug absorption. Intravenous artesunate, administered at 3 mg/kg per dose in line with national and WHO guidelines for older children, is commonly reported. Treatment duration typically ranges from 5 to 7 days, depending on clinical response and complications. In some cases, therapy is transitioned to oral artemether–lumefantrine after initial parenteral treatment [15].

Challenges related to antimalarial dosing in neonates are also evident. The lack of infant‐specific formulations increases the risk of underdosing or overdosing, a concern acknowledged by the WHO. Instances of inappropriate chloroquine dosing prior to referral have been documented [14, 17]. Moreover, drugs such as primaquine, which are standard in older children, are not recommended in neonates due to insufficient safety data and concerns related to breastfeeding and immature drug metabolism [21, 22]. Developmental differences in pharmacokinetics and pharmacodynamics further complicate treatment decisions in this age group.

Finally, the frequent initiation of empirical antibiotic therapy in reported cases reflects both the difficulty of distinguishing malaria from bacterial sepsis and the possibility of co‐infection. This practice underscores the need for improved diagnostic strategies and greater clinical awareness to ensure timely and appropriate management of neonatal malaria.

This study has several important limitations. Firstly, confirmation of congenital malaria requires testing of the mother, the newborn, and umbilical cord blood, with identification of the same *Plasmodium* species in all samples; however, most included studies did not consistently report these diagnostic criteria. Second, the review is limited to Ethiopia; broader geographic coverage could better reflect the level of attention given to this condition across endemic regions. Consequently, large‐scale, well‐designed studies are needed to generate more robust evidence on the burden, diagnosis, and outcomes of congenital and neonatal malaria.

## 6. Conclusion and Recommendations

*P. falciparum* is the most common cause of congenital and neonatal malaria reported in Ethiopia. Despite the country′s high malaria burden, these conditions remain underrecognized, with available evidence largely limited to case reports and small case series. Larger, well‐designed studies are needed to better define disease burden, clinical spectrum, and outcomes. Strengthening malaria prevention during pregnancy and improving neonatal diagnostic vigilance are essential to reduce associated morbidity and mortality.

**Nomenclature**
CM: congenital malaria
NM: neonatal malaria
PCR: polymerase chain reaction
WHO: World Health Organization

## Author Contributions

Yemane Leake: conceptualization, visualization, supervision, writing – original draft preparation. Hindeya Hailu Hagos: data curation, writing – original draft preparation. Birhanu Kassie Reta: visualization, investigation, writing – review and editing.

## Funding

No funding was received for this manuscript.

## Consent

The authors have nothing to report.

## Conflicts of Interest

The authors declare no conflicts of interest.

## Data Availability

The data that support the findings of this study are available on request from the corresponding author. The data are not publicly available due to privacy or ethical restrictions.
